# First Principles Study of the Photoelectric Properties of Alkaline Earth Metal (Be/Mg/Ca/Sr/Ba)-Doped Monolayers of MoS_2_

**DOI:** 10.3390/molecules28166122

**Published:** 2023-08-18

**Authors:** Li-Zhi Liu, Xian-Sheng Yu, Shao-Xia Wang, Li-Li Zhang, Xu-Cai Zhao, Bo-Cheng Lei, Hong-Mei Yin, Yi-Neng Huang

**Affiliations:** 1 Xinjiang Laboratory of Phase Transitions and Microstructures in Condensed Matter Physics, College of Physical Science and Technology, Yili Normal University, Yining 835000, China; liulizhi9902@sina.com (L.-Z.L.); yuxs997@sina.com (X.-S.Y.); lbc0428@sina.com (B.-C.L.); jisuan2000y@sohu.com (H.-M.Y.); ynhuang@nju.edu.cn (Y.-N.H.); 2 Physics and Electronic Engineering College, Kashi University, Kashi 844000, China; wwsx888666@sina.com; 3 National Laboratory of Solid State Microstructures, School of Physics, Nanjing University, Nanjing 210093, China

**Keywords:** alkaline earth metals, MoS_2_, first principles, electronic structure, optical properties

## Abstract

The energy band structure, density of states, and optical properties of monolayers of MoS_2_ doped with alkaline earth metals (Be/Mg/Ca/Sr/Ba) are systematically studied based on first principles. The results indicate that all the doped systems have a great potential to be formed and structurally stable. In comparison to monolayer MoS_2_, doping alkaline earth metals results in lattice distortions in the doped system. Therefore, the recombination of photogenerated hole–electron pairs is suppressed effectively. Simultaneously, the introduction of dopants reduces the band gap of the systems while creating impurity levels. Hence, the likelihood of electron transfer from the valence to the conduction band is enhanced, which means a reduction in the energy required for such a transfer. Moreover, doping monolayer MoS_2_ with alkaline earth metals increases the static dielectric constant and enhances its polarizability. Notably, the Sr–MoS_2_ system exhibits the highest value of static permittivity, demonstrating the strongest polarization capability. The doped systems exhibit a red-shifted absorption spectrum in the low-energy region. Consequently, the Be/Mg/Ca–MoS_2_ systems demonstrate superior visible absorption properties and a favorable band gap, indicating their potential as photo-catalysts for water splitting.

## 1. Introduction

Photocatalysts are frequently synthesized using materials with lower dimensions, such as graphene [1,2], hexagonal boron nitride [2], transition metal sulfides [3,4], and g-C_3_N_4_ [5], due to their high specific surface area, abundance of active catalytic sites, and high electron–hole separation rate [6,7]. Moreover, monolayer molybdenum disulfide (MoS_2_) is a widely researched excellent representative of two-dimensional (2D) materials with graphene-like structures. MoS_2_ is highly valued in the fields of photocatalysis, solar energy conversion, photovoltaics, and optoelectronics due to its optimal band gap, exceptional carrier mobility, and superior current-switching performance [8,9,10,11]. MoS_2_ is a layered material with an S–Mo–S sandwich structure, where a central layer of Mo atoms is flanked by two layers of S atoms above and below [12]. Monolayer MoS_2_ possesses a direct bandgap [13], enhancing its efficiency for water splitting. However, the efficient utilization of sunlight by 2D materials is limited by quantum size effects [14]. Substitution doping is an effective step to enhance the electronic properties of 2D materials’ device applications, similar to their use in conventional semiconductors [15,16,17,18,19]. Monolayer MoS_2_-doped Mn, Fe, Co, and Zn, for instance, as diluted magnetic semiconductors, could be applied in memory devices, store and read hard disks or MRAM [20,21]. P-type Au-doped MoS_2_ is applied in gas-sensing devices [22]. Re–MoS_2_, Mn–MoS_2_, Fe–MoS_2_ and Zn–MoS_2_ [16,23] have been used as n-type semiconductors, and Nb–MoS_2_ has been excellently applied in p-type semiconductors in optoelectronic devices, such as photodetectors and optical modulators, etc. The compounds mentioned in the above paragraph have all been synthesized experimentally. The experimental measurement analysis of the compounds has demonstrated that doping transition metal elements can induce a transformation from the intrinsic state to either n-type or p-type states in monolayer MoS_2_, which allows the noble compounds to be applied to different devices.

Moreover, the results of the first principles calculation and experimental test analyses of Co^2+^ (or Ni^2+^)-doped MoS_2_ all show that the introduction of Co^2+^ or Ni^2+^ results in the enhancement of monolayer MoS_2_ photocatalysis [24,25]. The prediction of the first principles computation of monolayer MoS_2_’s optical properties is consistent with experimental results. The conclusion indicates that doping impurities enhance monolayer MoS_2_ photocatalysis, and the first principles simulation method is a credible method.

The impurities mentioned in the above examples are transition metals. However, transition metal doping cannot reduce the bandgap to a lower width because of its local d orbital. The alkaline earth metals, including Be, Mg, Ca, Sr, Ba and others, constitute the second group of elements in the periodic table. In contrast to transition metal doping, alkaline earth metals do not involve localized d orbitals, so the doping of alkaline earth metals can effectively reduce the optical threshold energy of semiconductors [26]. The electronic structures of 2D materials with a graphene-like structure can be modified by the incorporation of alkaline earth metals. Studies conducted by Ullah [27] et al. and Tayyab M. [28] have demonstrated that doping graphene with Be and Mg can effectively modulate its band gap. Kumar G. M. [29] employed a hydrothermal method to synthesize Sn_0.98_Mg_0.02_S_2_ nanosheets and observed a red-shift in the absorption spectrum, indicating an enhanced absorption of visible light. Ani A. et al. [30] proposed that Ca-doped SnSe_2_ monolayers exhibit superior photocatalytic capacity. Doping graphene, or two-dimensional graphene-like materials, such as sulfur groups, with alkaline earth metals, has been demonstrated to induce minor defects that impact the electronic structure. This ultimately leads to an increase in carrier mobility and the improvement of absorption in visible light. It is postulated that the incorporation of alkaline earth metals can effectively modulate the photoelectric properties of monolayer MoS_2_. However, a systematic investigation into the impact of alkaline earth metals on the optoelectronic properties of monolayer MoS_2_ has yet to be conducted. Therefore, it is worthwhile to delve deeply into the mechanisms and regulatory laws governing its stable existence.

To sum up, monolayer MoS_2_, a semiconductor material, has been widely studied and applied, but the effective utilization of sunlight is limited by its electronic mobility, which is restricted to the finite sizes of the synthesized monolayer MoS_2_. It is found that the doping of alkaline earth metals (Be/Mg/Ca/Sr/Ba) can change the electronic band structure of the pristine materials and affect the transport of electrons. However, the effect of the alkaline earth metal doping of MoS_2_ on the photoelectric performance of the system has not been systematically theoretically studied. In our work, the energy band structure, density of states, and optical properties of monolayer MoS_2_ doped with alkaline earth metals were systematically studied based on first principles. The results show that the Be/Mg/Ca-MoS_2_ systems had superior visible absorption performance and ideal band gaps, and the potential to be used as photocatalyst candidates for water splitting.

## 2. Results

### 2.1. Analysis of Lattice Constants and Bond Population

The GGA-PBE calculation method was used to optimize the systems’ geometry. The lattice constants optimized were a = b = 3.17 and c = 12.43; the errors relative to the experimental values [31] and those of Hu [32] et al. were within 1%, which indicates that the computational methods and parameters used in this paper are reliable. Table 1 summarizes the optimized crystal structures of monolayer MoS_2_ and X-MoS_2_ systems. The formation energy of the doped system is an indicator of the degree of formation difficulty. A lower formation energy implies that the doped system more easily forms. The formation energy is calculated as:

(1) $$E_{f}=E_{doped}-E_{undoped}+n\mu_{Mo}-n\mu_{X}$$

$E_{doped}$ and $E_{undoped}$ are the total energy of doped and undoped MoS_2_, $\mu_{Mo}$ and $\mu_{X}$ are the chemical potential of the Mo and X atoms, while *n* denotes the number of X atoms doped into the supercell [10]. As shown in Table 1, the formation energy of the doped systems decreased gradually with the decrease in the doped atoms’ radius (*R_Ba_* > *R_Sr_* > *R_Ca_* > *R_Mg_*), which suggest that the smaller the atomic radius of the doped system was, the more easily the system formed. In Table 1, the absolute value of the formation energy of all the doped systems is shown within a reasonable range. The compounds with an absolute value of formation energy in that range can be synthesized via experiments [33,34,35,36,37]. Therefore, it can be inferred that all the doped systems can be synthesized. The Mg–MoS_2_ system exhibited the lowest formation energy among the five, indicating its superior suitability to formation.

According to reference [38], the covalent properties of a chemical bond become stronger as the value of the bond population increases. The results presented in Table 1 demonstrate that the maximum population of Mo–S bonds in each doped system surpassed that of the monolayer MoS_2_ system, while the minimum Mo–S bonding population in all doping systems was lower than that of the intrinsic system. However, the maximum value varied much more than the minimum value, which demonstrates that the stability of the doped systems was not destroyed by the introduction of impurity atoms. 

The higher the Δγ value, the more pronounced the distortion of the corresponding system becomes [39]. In the table, we can see that the Δγ values of all doped systems were greater than that of a pure one, which implies that all the systems were distorted, and this aligns with the conclusion that the band gaps of the doped monolayer MoS_2_ were reduced. Moreover, Ba–MoS_2_ had the greatest degree distortion, which corresponds to its having the smallest bap gap. The optimized Mo–S bond length of 2.41 coheres with the one Li et al. reported [40]. It was observed that the Δγ values of the doped systems, except Be–MoS_2_, increased as the radius of the dopant atom increased. It was suggested that the degree of lattice distortion also increased with the dopant atom’s radius. The lattice distortion caused a separation between the positive and negative charge centers within the defect, which resulted in the creation of an internal electric dipole moment and a local potential difference. The introduction of alkaline earth metal elements was found to increase the rate of separation of photogenerated electron–hole pairs and decrease the energy needed for electron leap, as reported in reference [41]. 

**Figure 1:**
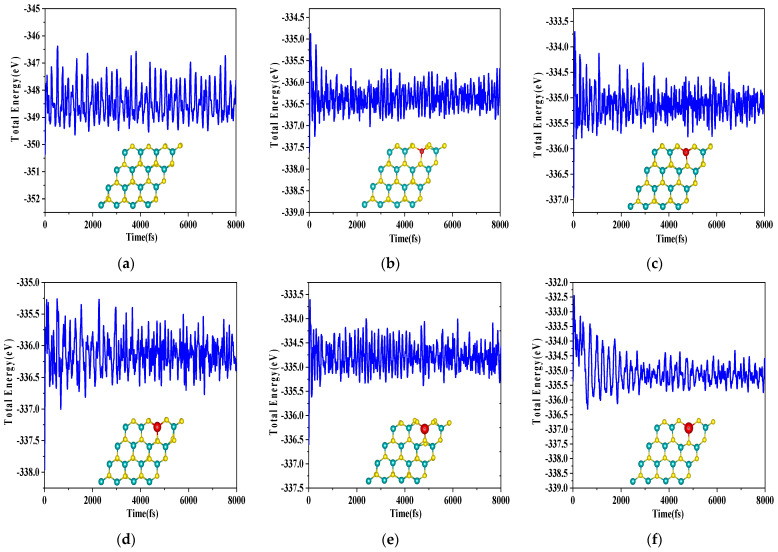
AIMD (300 K) of MoS_2_ systems pre- and post-doping: (**a**) monolayer MoS_2_; (**b**) Be–MoS_2_; (**c**) Mg–MoS_2_; (**d**) Ca–MoS_2_; (**e**) Sr–MoS_2_; (**f**) Ba–MoS_2._ (yellow, blue and red colors represents S, Mo and X, respectively).

In order to further verify its thermal stability, the AIMD (Ab Initio Molecular Dynamics) [42] analysis of monolayer MoS_2_ molybdenum and X–MoS_2_ systems was performed, and the temperature was set to room temperature (300 K), as shown in Figure 1. It can be seen that after 8000 steps of AIMD, with a time step of 1 fs, the structures of the six systems remained stable—there was no bond breaking, and the energy fluctuation was very small. The results show that the monolayer MoS_2_ and X-MoS_2_ systems have high stability at room temperature (300 K) [43,44].

### 2.2. Band Structures

**Figure 2:**
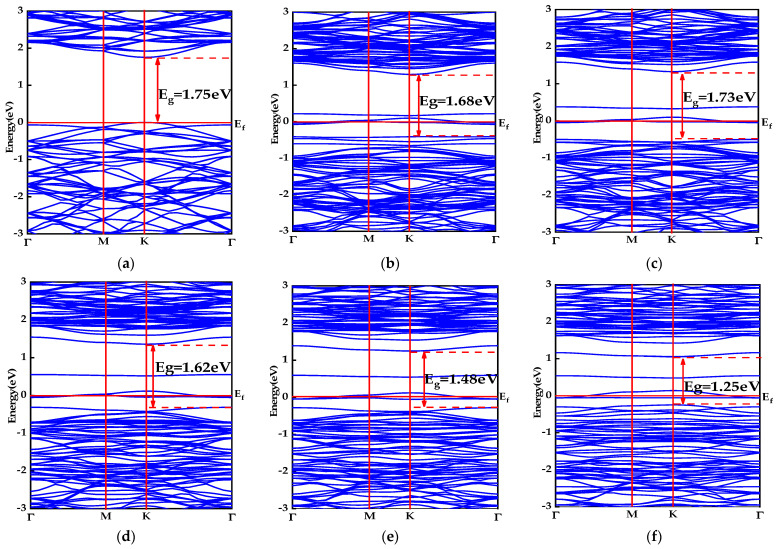
Band structures of MoS_2_ systems pre- and post-doping: (**a**) monolayer MoS_2_; (**b**) Be–MoS_2_; (**c**) Mg–MoS_2_; (**d**) Ca–MoS_2_; (**e**) Sr–MoS_2_; (**f**) Ba–MoS_2_.

The band structures of the monolayer MoS_2_ and X–MoS_2_ systems are presented in Figure 2. The Fermi energy level was set at 0 eV, and the energy range of −3~3 eV was selected for comparative analysis. The band structure of the monolayer MoS_2_ system is illustrated in Figure 2a; the conduction band minimum (CBM) and valence band maximum (VBM) are both located at the high symmetry point K. The semiconductor exhibited a direct band gap of 1.75 eV. This value was only 0.57% off from the experimental value of 1.74 eV [45], and was similar to the calculated value of 1.78 eV found in the literature [46].

Figure 2b–f illustrates the band structure of MoS_2_ doped with alkaline earth metals in a clear and concise manner, providing valuable insights into its electronic properties. The CBM and VBM of the doped systems exhibited a downward shift in energy compared to those of the monolayer MoS_2_. The X–MoS_2_ systems had band gaps of 1.68, 1.73, 1.62, 1.48, and 1.25 eV, which were observed to be reduced as the atomic numbers of doped alkali earth metal elements increased, except for Be. The decrease in the band gap indicated an effective reduction in the energy required for electron transition, thereby enhancing electron mobility. The doped systems displayed a series of impurity energy levels that were relatively independent, acting as an intermediary for valence band electrons to transition into the conduction band and enhancing the probability of electron transition to the conduction band. The doped systems were found to possess an enhanced optical absorption capacity. Notably, Ba–MoS_2_ exhibited the smallest direct band gap, indicating a minimal energy requirement for electron transition in this system. Therefore, it can be inferred that Ba–MoS_2_ boasts superior optical absorption capabilities. 

In the doped systems exposed to visible light, valence electrons were induced to transition between valence and impurity levels as well as conduction bands, leading to the generation of photonic electron–hole pairs. The relatively independent impurity levels effectively reduced the probability of the electron–hole pairs’ recombination, and enhanced optical absorption capacity.

### 2.3. Density of States

**Figure 3:**
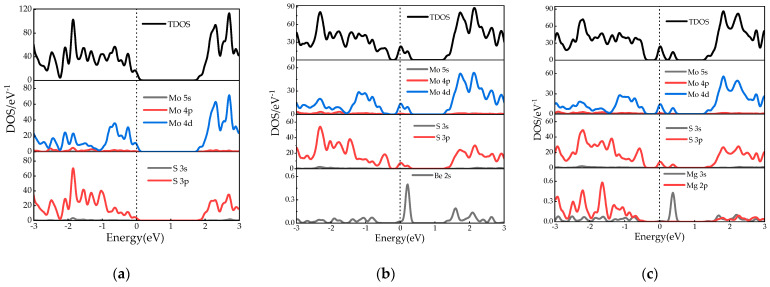

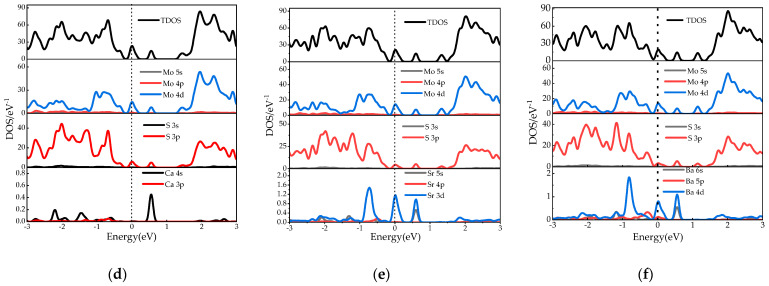
Density of states of MoS_2_ systems pre- and post-doping: (**a**) monolayer MoS_2_; (**b**) Be–MoS_2_; (**c**) Mg–MoS_2_; (**d**) Ca–MoS_2_; (**e**) Sr–MoS_2_; (**f**) Ba–MoS_2_.

The density of state (DOS) plots for monolayer MoS_2_ and X–MoS_2_ systems are presented in Figure 3, encompassing energies ranging from −3 to 3 eV. In Figure 3a, the densities of states in a monolayer MoS_2_ are depicted, wherein the conduction band is dominated by Mo–4d, Mo–5s, and S–3p states, and the valence band is dominated by Mo–4d, S–3s, and S–3p states. Both the CBM and VBM were mainly determined by the Mo–4d and S–3p states, which is consistent with what has been found in the literature [10].

Figure 3b–f display the DOS plots of the MoS_2_ systems doped with Be, Mg, Ca, Sr, and Ba, respectively. As illustrated in Figure 3b–d, in the Be/Mg/Ca-doped systems, the valence bands were mainly composed of Mo–4d and S–3p. The CBM was primarily derived from Mo–4d and S–3p states, with minor contributions from Be–2s, Mg–2p, Mg–3s, and Ca–4s states. The Ca–4s state had almost no effect on the conduction band. The Be–2s, Mg–3s, and Ca–4s states led to impurity levels near and above the Fermi level. The band gaps of the materials were narrowed due to impurity doping, which is consistent with the result regarding band structure. The DOS values of the Sr/Ba-doped MoS_2_ systems reveal that the effects of Sr–3d and Ba–3d states on the conduction band were more significant. The introduction of impurities into the materials led to a reduction in the band gaps, which is consistent with the results obtained from band structure analysis. The valence bands of the Sr/Ba-doped MoS_2_ systems were determined by the Mo–4d, S–3p, and S–3s states, while the Ba–5p state manifested a weak peak in the lower valence band of the Ba–MoS_2_ system, similarly to the results of other doped systems. The band gap of the Ba-doped system is the smallest. In Section 2.1, we can see that the Δγ value of Ba–MoS_2_ was the smallest, which demonstrates that Ba–MoS_2_ manifested the greatest degree of lattice distortion. This is why Ba–MoS_2_ had the smallest bandgap [10].

The contributions of S or d-state electrons from doped elements near and above the Fermi level are particularly obvious; they determine the impurity level, which causes the conduction band minimum (CBM) and valence band maximum (VBM) in the doped systems to shift down significantly, reducing the band gap, and ultimately leading to a decrease in photon excitation energy and a red shift in the predicted absorption spectrum of the doped systems.

**Figure 4:**
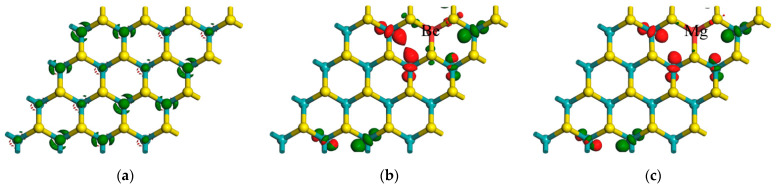

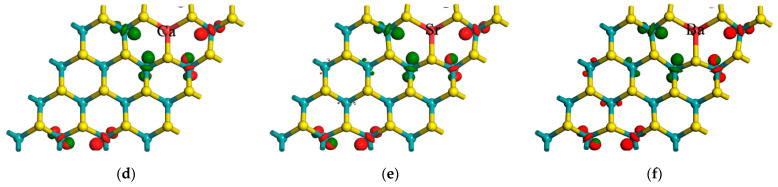
LUMO and HOMO of MoS_2_ systems before and after doping: (**a**) LUMO and HOMO of monolayer MoS_2_; (**b**) Be–MoS_2_; (**c**) Mg–MoS_2_; (**d**) Ca–MoS_2_; (**e**) Sr–MoS_2_; (**f**) Ba–MoS_2_ (red and green filled areas indicate LUMO and HOMO, respectively).

To investigate the microscopic changes in energy levels of each system, we created LUMO (Lowest Unoccupied Molecular Orbital) and HOMO (Highest Occupied Molecular Orbital) diagrams for the monolayer MoS_2_ and X–MoS_2_ systems. Figure 4 shows the LUMO and HOMO, represented by the red and green areas, respectively. The isosurface was set as 0.04 e/Å^3^. The larger the area, the greater its ability to gain (lose) electrons. The LUMO orbitals possessing lower energy levels were more conducive to the obtaining of electrons, whereas the HOMO orbitals with higher electron energies were more likely to show electron loss [47]. In Figure 4a, we can see that the LUMO and HOMO of the monolayer MoS_2_ were distributed symmetrically, indicating that the centers of the positive and negative charges were in the same position, and there was no internal electric field. According to Figure 4b–f, compared to the monolayer MoS_2_, the LUMO and HOMO maps of the doped systems were redistributed, mostly clustered around the Mo and S atoms near the dopant atoms. In Figure 4b, the area of the HOMO of Mo is greater than that of the LUMO of S atoms near the Be atom, indicating that Mo and S lost and gained electrons, respectively, and electrons were easily transferred from the Mo atom to the S atom. In Figure 4c, we can see that the Mo atoms of the Mg–MoS_2_ had the greatest ability to gain electrons, while in the Ca–MoS_2_ system, Mo and S showed almost equal electron gain and loss. The Mo atoms in the Sr–MoS_2_ and Ba–MoS_2_ systems showed the greatest electron loss. The observed charge transfer and asymmetric distribution are in agreement with the findings of the static dielectric constant below.

### 2.4. Work Functions

**Figure 5:**
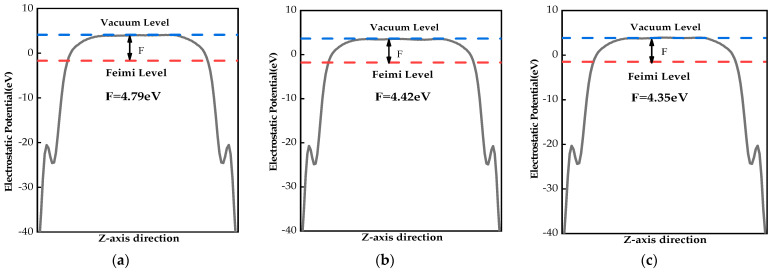

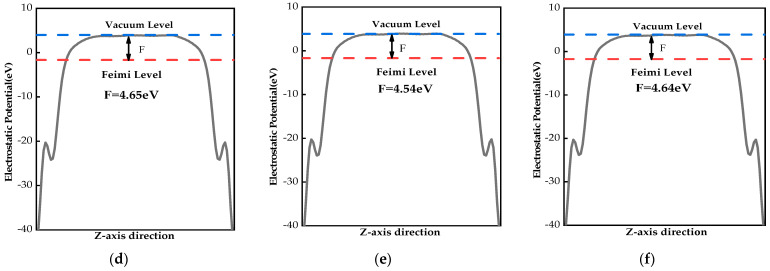
Work functions of MoS_2_ systems before and after doping: (**a**) monolayer MoS_2_; (**b**) Be–MoS_2_; (**c**) Mg–MoS_2_; (**d**) Ca–MoS_2_; (**e**) Sr–MoS_2_; (**f**) Ba–MoS_2_.

To investigate charge transfer in monolayer MoS_2_ before and after doping, the work functions of monolayer MoS_2_ and X–MoS_2_ systems were calculated. The work function, as illustrated in Formula (2), is defined as the minimum energy required for an electron to transition from the Fermi energy level to the vacuum layer [48].

(2) $${{\Phi =E}_{vac}-E}_{F}$$

where Φ represents work function, $E_{vac}$ is vacuum level, and $E_{F}$ denotes Fermi level. The work function of the monolayer MoS_2_ was 4.79 eV, as shown in Figure 5a, which is consistent with the value of 4.89 eV calculated by Wei [49]. The Be/Mg/Ca/Sr/Ba-doped systems had lower work functions than that of the monolayer MoS_2_ (4.42, 4.35, 4.65, 4.54, and 4.64 eV, respectively). It was shown that doping with alkaline earth metals effectively reduced the energy required for electrons to escape from the interior to the semiconductor surface. Hence, the doped systems exhibited a better capacity for electron transition.

### 2.5. Optical Properties

**Figure 6:**
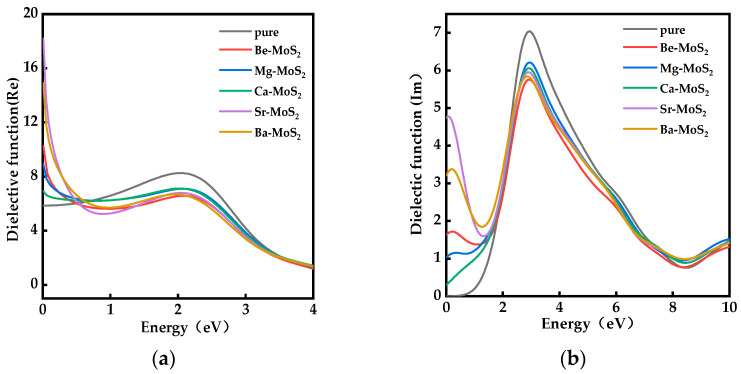
Dielectric function diagram of MoS_2_ systems before and after doping: (**a**) real part and (**b**) imaginary part.

Figure 6a shows the real part of the dielectric function (ε_1_) of monolayer MoS_2_ and X–MoS_2_ systems. The real part reflects the polarization strength of the semiconductor material in the presence of an external electric field with a change in incident photon energy. A higher value of ε_1_ indicates an enhancement in the capacity of the systems to bind charges, thereby increasing their polarizability [50]. When the incident photon energy was 0 eV, the systems exhibited a static dielectric constant (ε_0_). From Figure 6a, we can see that the ε_0_ values of the monolayer MoS_2_ and the doped systems were 5.88, 10.31, 8.89, 7.05, 18.20, and 14.94, respectively. In comparison to the monolayer MoS_2_, all the doped systems showed an increase in values of ε_0_, indicating an enhancement in the polarizability of the X–MoS_2_ systems. Therefore, in doped systems, the migration and separation rates of joint electron–hole pairs were accelerated, leading to an improvement in the optical performance of the system. The Sr–MoS_2_ system exhibited the highest values, indicating that it possessed the strongest polarization, excitation strength, and charge binding capability. 

In Figure 6b, the imaginary parts of the dielectric function (ε_2_) of the monolayer MoS_2_ and doped systems are shown, varying with incident photon energy. The larger the values of ε_2_, the greater the probability of photon absorption, and the stronger the absorption energy of the materials. The value of ε_2_ is dependent on the number of electrons in the excited state, which increases the likelihood of the next transition occurring [50]. It can be observed that the ε_2_ of the monolayer MoS_2_ displayed a dielectric absorption peak at approximately 2.90 eV. This peak can be primarily attributed to the interband transition between the S–3p and Mo–4d states of electrons. The dielectric peaks of ε_2_ in the X–MoS_2_ systems were shifted towards the low-energy region compared to the monolayer MoS_2_. In the low-energy region, the intensity of the imaginary peak was significantly higher in the doped systems than in the intrinsic system. Because of a higher number of electrons present in the impurities formed by the doped system near the Fermi level, an enhancement in the light absorption of the doped systems in the low-energy region was observed. In the X–MoS_2_ systems, ε_2_ exhibited a significant response at a photon energy of 0 eV due to the partial occupation of energy levels of dopant atoms and the impurity energy levels straddling the Fermi energy levels. The presence of dopant elements facilitated electron transfer between multiple degenerate impurity levels, thereby enhancing the low-energy photon absorption of MoS_2_.

**Figure 7:**
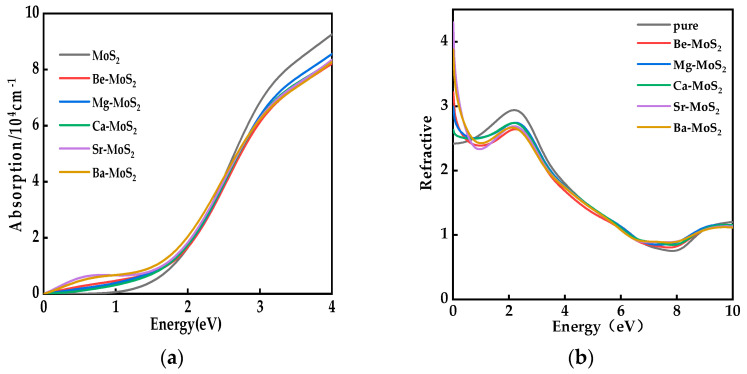
(**a**) Light absorption diagram, (**b**) refractive index of MoS_2_ systems before and after doping.

The absorption spectra and refractive indices of monolayer MoS_2_ and X–MoS_2_ systems are presented in Figure 7. As depicted in Figure 7a, in comparison to the monolayer MoS_2_, the doped systems displayed notable redshifts, and their light responses were extended to the low-energy region. Therefore, the doped systems showed a better light absorption capacity. The Sr–MoS_2_ and Ba–MoS_2_ systems displayed the same absorption peaks in the low-energy region (0–1.6 eV), demonstrating the two systems had strong capacity to use visible light. 

Based on the refractive index curves depicted in Figure 7b, it can be inferred that the static refractive index of the monolayer MoS_2_ was 2.452 when the photon incident energy was 0 eV. This value is consistent with the theoretical value of 2.475 [51]. After doping, the static refractive index values of the X–MoS_2_ systems were all greater than that of the pure system. However, with the increase in incidence photon energy, the refractive indices of all systems tended to overlap and approach unity, which indicates that the monolayer MoS_2_ remained transparent both before and after doping.

### 2.6. Band Alignment

**Figure 8:**
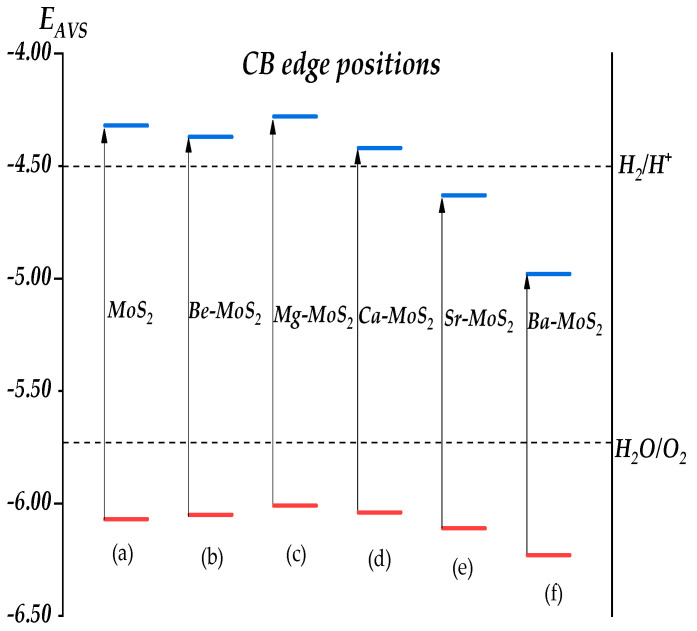
Band edge alignments of MoS_2_ systems before and after doping (redox potentials of H_2_/H^+^and H_2_O/O_2_ at pH=0 were used as standards). (a) monolayer MoS_2_; (b) Be–MoS_2_; (c) Mg–MoS_2_; (d) Ca–MoS_2_; (e) Sr–MoS_2_; (f) Ba–MoS_2_.

To understand the effects of alkaline earth metal doping on the photocatalytic capacity of the monolayer MoS_2_, the band alignments of both the monolayer MoS_2_ and X–MoS_2_ systems were calculated, and are illustrated in Figure 8. We used the concept of semiconductor electronegativity [52] to determine the redox and oxidation potentials for each water system. The position of the band edge was calculated using the Milligan electronegativity equation, which is illustrated in [53,54,55].

(3) $$E_{VBM}=\chi -E_{elec}+0.5E_{g}$$

(4) $$E_{CBM}=E_{VBM}-E_{g}$$

In this equation, χ represents the geometric mean of the Milligan electronegativity of all atoms, $E_{elec}$ represents the free electron energy of 4.5 eV on the standard hydrogen electrode scale, and $E_{g}$ represents the semiconductor band gap [56]. This method did not provide exact values, but enabled a general evaluation of each system’s position on the normal hydrogen electrode scale. The band edges of monolayer MoS_2_ were suitable for a semiconductor photocatalyst, and this is consistent with experimental results [57]. Yet the oxidation and reduction potential of Sr/Ba–MoS_2_ systems were too low to generate a sufficient driving force for O_2_ and H_2_ production compared to standard water-splitting redox potential. It was suggested that the Sr/Ba–MoS_2_ systems needed to elevate their CBM and VBM positions relative to the water splitting potential. The Be–MoS_2_, Mg–MoS_2_, and Ca–MoS_2_ systems all had band gaps that straddled the redox potential of water. However, the CBM of Ca–MoS_2_ was found in close proximity to the H_2_/H^+^ reduction potential, rendering its reduction capacity weaker than that of the Be/Mg/Ca–MoS_2_ systems. Hence, the Be/Mg/Ca–MoS_2_ systems possessed suitable band edge positions, making them promising candidates for high-performance photocatalysts. In comparison, Sr/Ba–MoS_2_ systems were not suitable for water splitting. However, the Sr/Ba–MoS_2_ system had higher work functions and smaller band gaps, which demonstrates that Sr/Ba–MoS_2_ could potentially eliminate the Schotk barrier height of MoS_2_-based transistors and reduce the contact resistance. Schottky contact is one of the bottlenecks currently limiting the application of TMDs (Transition Metal Dichalcogenides) in semiconductors [58,59,60]. The significant reduction in the conduction band of the Sr/Ba–MoS_2_ system means that metal–MoS_2_ doped with Sr/Ba has the capacity to reduce the Schottky barrier, which thus establishes a new method for the modulation of Schottky barrier height [59]. In the future, we will study Sr/Ba–MoS_2_ contact.

## 3. Experimental Section

**Figure 9:**
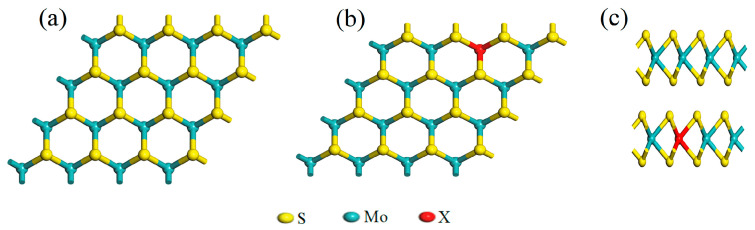
MoS_2_ supercell model both pre- and post-doping: (**a**) top view of monolayer MoS_2_; (**b**) top view of X-MoS_2_; (**c**) side view of monolayer MoS_2_ and X-MoS_2_.

It is worth noting that the crystal structure of MoS_2_ can adopt various phases, including 1T, 2H, and 3R phases, depending upon its arrangement. In this paper, we have opted for the stable 2H-MoS_2_ [61] with space group P63/mc [62] as the basis to construct a monolayer MoS_2_ supercell of 4 × 4 × 1. This supercell contained a total of 48 atoms, including 16 Mo atoms and 32 S atoms. In Figure 9, the monolayer MoS_2_ is shown with an alkaline earth metal atom X (X = Be/Mg/Ca/Sr/Ba) replacing one Mo atom. The supercell system’s geometry was optimized both pre- and post-doping, with properties calculated using single-point energy after optimization. The calculations involved the following valence electronic arrangements: Mo 4d^5^5s^1^, S 3s^2^3p^4^, Be 2s^2^, Mg 3s^2^2p^6^, Ca 4s^2^3p^6^, Sr 5s^2^4p^6^3d^10^, and Ba 6s^2^5p^6^4d^10^.

This study employed the CASTEP software package [63,64] and utilized the projected augmented wave (PAW) method within a density functional theory framework to conduct computational simulations. The PAW method was employed to depict the impact of the ionic reality on the valence electrons, while the exchange–correlation interaction was characterized by employing the Perdew–Burke–Ernzerhof (PBE) [65] test within the generalized gradient approximation (GGA). In this study, we utilized the semi-empirical Tkatchenko–Scheffler (TS) dispersion correction method to fine-tune van der Waals forces. To mitigate interlayer coupling effects, we introduced a vacuum layer with a thickness of 20 Å in the c-direction. We accorded convergence tests and employed the BFGS algorithm for both geometry optimization and electronic calculation; the cutoff energy for the plane–wave basis was set as 450 eV [65], according to the Monkhorst–Pack scheme [31], and the k grid was selected as 5 × 5 × 1 (detailed information of convergence tests is reported in Figure S1 of Supplementary Materials). The self-consistent precision SCF was set as 2.0 × 10^−6^ eV/atom, and the maximum stress was set as 0.1 GPa. The maximum displacement was less than 2 × 10^−3^ Å and the convergence accuracy of the interatomic force field was 0.05 eV/Å. 

## 4. Conclusions

The electronic structures and optical properties of the MoS_2_ systems were studied. The authors employed first principles based on density function theory to investigate the effects of doping alkaline earth metals into monolayer MoS_2_. Following analyses of the results regarding AIMD and bond population, it was found that all impurity systems exhibited thermodynamic stability. The formation energy’s numerical value also suggests the potential for formation in all impurity systems, with Mg–MoS_2_ exhibiting the highest propensity for formation. The Δγ values indicate that all impurity systems will show different degrees of lattice distortion, resulting in a reduced band gap width compared to pure systems. Ba–MoS_2_ showed the greatest degree of distortion, so its band gap width was also the narrowest. The introduction of impurities leads to the emergence of impurity levels in close proximity to the Fermi level, which are clearly visible in the band structures of all impurity systems, thereby facilitating electron transitions and promoting the generation of photo-generated electron–hole pairs. In the analysis of the work function, it was observed that the impurity systems exhibited lower values compared to pure systems, indicating that the introduction of impurities enhances electron transfer from the interior of the system to its surface, facilitating their participation in photocatalytic reactions. The static dielectric function values of impure systems are higher than those of pure systems, indicating that the introduction of impurities generates a polarizing electric field and impedes the recombination of photo-generated electron–hole pairs. According to the analysis of the absorption spectra, it is evident that all impurity systems exhibit a red shift in comparison to the pure system, indicating an expanded range of response to solar light upon the introduction of impurities. This expansion has proven advantageous for enhancing photocatalytic capabilities. From the band alignment analysis, we can conclude that Be/Mg/Ca–MoS_2_ systems possess suitable band edge positions, making them promising candidates for use as high-performance photocatalysts. In comparison, the Sr/Ba–MoS_2_ systems were not suitable for water splitting. However, the Sr/Ba–MoS_2_ system showed higher work functions and smaller band gaps, which demonstrates that Sr/Ba–MoS_2_ could potentially reduce the height of the Schotk barrier in MoS_2_-based transistors, and reduce the contact resistance. Sr/Ba–MoS_2_ contact is a new approach to the modulation of Schottky barrier height. In the future, we will study Sr/Ba–MoS_2_ contact.

## Figures and Tables

**Table 1:** Formation energy, bond population and bond length of MoS_2_ systems pre- and post-doping.

	Formation Energy/eV	Bond Population of Mo–S (Max)	Bond Population of Mo–S (Min)	Bond Length of Mo–S/Å (Min)	Bond Length of Mo–S/Å (Min)	^1^ Δγ/Å
MoS_2_	/	0.37	0.37	2.4083	2.4077	0.0006
Be-MoS_2_	10.92	0.52	0.34	2.4274	2.3369	0.0905
Mg-MoS_2_	7.27	0.47	0.36	2.4291	2.3960	0.0331
Ca-MoS_2_	12.17	0.50	0.34	2.4461	2.3901	0.0560
Sr-MoS_2_	13.45	0.49	0.35	2.4593	2.3841	0.0752
Ba-MoS_2_	14.60	0.47	0.34	2.4750	2.3775	0.0975

^1^ Δγ is the difference between the maximum and minimum Mo–S bond lengths.

## Data Availability

The data preesented in this syudy are available on request from the corresponding authors.

## Supplementary Materials

The following supporting information can be downloaded at: https://www.mdpi.com/article/10.3390/molecules28166122/s1, Figure S1: Convergence tests of MoS_2_.
