# Supplementary material for: First Principles Study of the Photoelectric Properties of Alkaline Earth Metal (Be/Mg/Ca/Sr/Ba)-Doped Monolayers of MoS2
Source: Molecules. 2023 Aug 18;28(16):6122. doi: 10.3390/molecules28166122 (PMC10458419; doi:10.3390/molecules28166122)
Supplement: Supplementary file 1 [file molecules-28-06122-s001.zip › molecules-2455408-supplementary.pdf]

Supplementary Material

First principles study of the photoelectric properties of alkaline earth metal (Be/Mg/Ca/Sr/Ba)-doped monolayers of MoS_2

Li-Zhi Liu, Xian-Sheng Yu, Shao-Xia Wang, Li-Li Zhang, Xu-Cai Zhao, Bo-Cheng Lei and Hong-Mei Yin, Yi-Neng Huang

1. Convergence test

In order to greatly reduce the calculation time on the basis of accurate calculation, convergence tests were carried out on the supercell size of MoS_2, cutoff energy of geometric optimization calculation and K-lattice points, as shown in Figure 1 (a), (b) and (c) respectively. As can be seen from Figure 1 (a), the supercell starts from 2×2×1, the energy of the system decreases somewhat and tends to be gentle. However, considering the large supercell energy is closer to the properties of the materials under study, 4×4×1 MoS_2 was selected as the research object on the basis of stable energy and sufficient computing resources. As can be seen from Figure 1 (b) and (c), when the truncation energy and K grid points were 450 eV and 5×5×1, the system energy decreases somewhat and tends to be flat. Therefore,we have used a cutoff energy of 450 eV and 5×5×1 K-points grid for all the calculations.

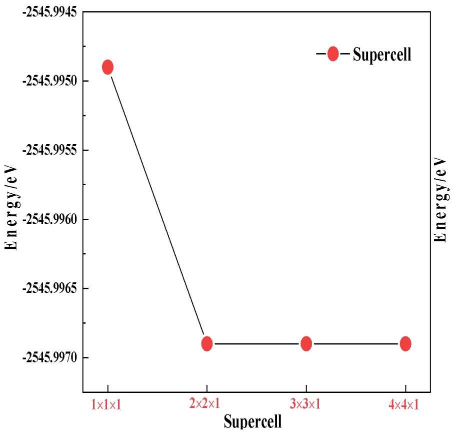

(a)

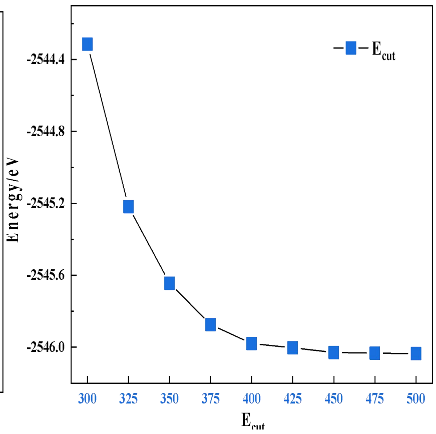

(b)

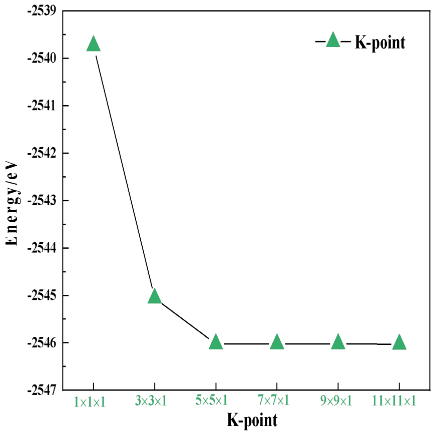

(c)

Figure S1 Convergence tests of MoS_2

(a) Convergence test of supercell; (b) Convergence test of cutoff energy; (c) Convergence test of K-point;
